# Advancing non-sterile pediatric pharmacotherapy through automated compounding and 3D printing of ondansetron hcl dihydrate dosage forms

**DOI:** 10.1177/10781552251383794

**Published:** 2025-10-25

**Authors:** Mahsa Bahman, M. Brooke Bernhardt, Cynthia A. Brasher, Julius Lahtinen, Sari Airaksinen, Niklas Sandler Topelius

**Affiliations:** 1 Pharmaceutical Sciences Laboratory, 278232 Åbo Akademi University, Turku, Finland; 2 CurifyLabs Oy, Helsinki, Finland; 3 Department of Pharmacy and Pharmaceutical Sciences, 5417 St Jude Children's Research Hospital, Memphis, Tennessee, USA

**Keywords:** Personalized medicine, semi-solid extrusion (SSE) 3D printing, ondansetron HCl dihydrate, blend homogeneity, quality control, orodispersible film (ODF)

## Abstract

**Background:**

Pediatric patients often require individualized medication dosing due to variations in age, weight, and swallowing ability. Commercial ondansetron HCl dihydrate formulations are limited by rigid dosing options, unpleasant taste (dysgeusia), and challenges with administration, which can result in poor adherence. Pharmaceutical compounding technologies, particularly those incorporating automation, offer a pathway toward patient-centric solutions. Semi-solid extrusion (SSE) 3D printing enables the controlled production of customized dosage forms.

**Methods:**

This study investigated the development and quality performance of personalized ondansetron HCl dihydrate dosage forms produced using an SSE-based automated compounding system. Three dosage forms (semi-solid gel tablet, anhydrous troche, and orodispersible film (ODF)) were formulated in various ondansetron concentrations: 0.81–7.5 mg. Formulations were developed and printed at a hospital setting demonstrating real-life hospital pharmacy conditions. Quality evaluations followed United States Pharmacopeia (USP) guidelines.

**Results:**

The printed ondansetron HCl dihydrate formulations met USP acceptance criteria for mass and content uniformity, syringe homogeneity, visual inspection, chemical stability, and in vitro dissolution performance. ODFs demonstrated rapid disintegration and are particularly suited for pediatric use. All dosage forms were reproducibly manufactured using the automated SSE printing platform, supporting flexible, on-demand production tailored to patient needs.

**Conclusion:**

SSE 3D printing offers a reliable and scalable compounding approach for producing personalized ondansetron HCl dihydrate formulations in hospital pharmacies. By integrating automation and digital manufacturing into pharmaceutical workflows, this study demonstrates the potential of next generation compounding systems to enhance dosing precision, regulatory compliance, and patient adherence in pediatric pharmacotherapy.

## Introduction

Pharmaceutical non-sterile compounding is undergoing a significant transformation as emerging technologies and digital workflows are introduced to meet the increasing demand for individualized therapies.^1^ While manual compounding remains common, modern approaches such as automated systems and 3D printing enable precise customization of medications particularly important in pediatric care, where standard commercial products often fail to meet age-specific dosing, swallowability mouthfeel, and adherence needs.^2–6^

Among these emerging technologies, semi-solid extrusion (SSE) based 3D printing or dispensing has demonstrated substantial potential in transforming drug production from a ‘one size fits all’ approach to one that is fully tailored to patient-specific needs. SSE enables volumetric, gravimetric or layer-by-layer deposition of gel- or paste-like materials to fabricate personalized dosage forms. Its low-temperature operation makes it particularly suitable for heat-sensitive active pharmaceutical ingredients (APIs). These methods offer tight control overdose accuracy, shape, and drug release characteristics, making it well-suited for pediatric patients requiring flexible dosing.^7,8^

Despite the widespread clinical use of ondansetron HCl dihydrate for preventing nausea and vomiting associated with chemotherapy, radiotherapy, or surgery,^9^ commercially available formulations may present challenges in pediatric settings. Although ondansetron is available in several dosage forms, they are not routinely available in all countries around the world. Dysgeusia (unpleasant taste), oral aversion, and difficulty swallowing solid dosage forms can lead to poor treatment adherence and increased risk of underdosing. These issues highlight the urgent need for more acceptable, individualized formulations for pediatric populations.

Orodispersible films (ODFs), chewable gel tablets, and anhydrous troche have emerged as patient-centric alternatives to conventional pediatric formulations such as suspensions and solutions, which are prone to dosing errors both in hospitals and at home. ODFs offer several advantages: they dissolve rapidly in the mouth without the need for water or chewing, improve patient compliance, and provide dose flexibility, an especially important factor in pediatric care^10–12.^ Furthermore, for drugs like ondansetron HCl dihydrate classified as BCS (Biopharmaceutics Classification System) Class II (low solubility, high permeability), ODFs can enhance solubility and bioavailability by facilitating rapid disintegration and mucosal absorption.^13^

While automation has long been adopted in sterile compounding, non-sterile compounding, especially for oral pediatric medications, has remained largely manual, labor-intensive, and error prone. This reliance on traditional methods contributes to variability in dose preparation and places a high burden on pharmacy staff. Emerging technologies such as 2D and 3D printing are beginning to bridge this gap by offering scalable, reproducible, and digitally traceable solutions. By integrating these technologies into hospital pharmacy workflows, it becomes feasible to produce individualized medications on demand while ensuring regulatory compliance, quality assurance, and workflow efficiency.^1^

The purpose of this study was to examine the application of SSE 3D printing within an automated compounding system (CSS) for producing three different pediatric-friendly ondansetron HCl dihydrate formulations: semi-solid gel tablets, anhydrous solid troche and ODFs all tailored in multiple dosages for flexible administration. The formulations were first developed within the lab and then recipes were transferred and printed at a hospital setting, highlighting a clinical collaboration aimed at translating digital compounding innovation into real-world pediatric pharmacotherapy.

## Materials & methods

To evaluate the reliability of the 3D-printed formulations, comprehensive quality assessments were conducted in accordance with the United States Pharmacopeia (USP). These included in-process controls, evaluation of formulation blend homogeneity, printing accuracy, mass and content uniformity, chemical stability, and in vitro dissolution testing. This study also incorporated high-performance liquid chromatography (HPLC) analysis for drug content verification, further supporting the consistency and quality of the formulations.

### Drug formulation development

**Figure 1.**
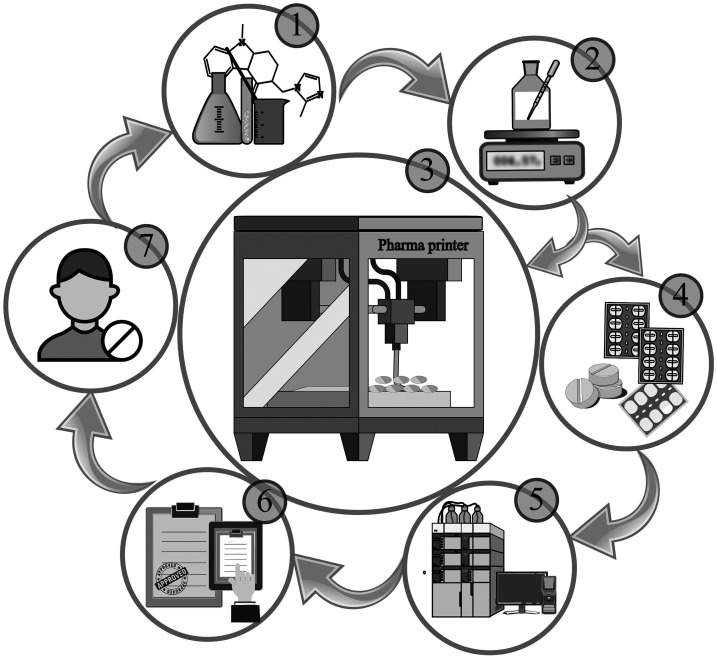
Workflow of the compounding system solution (CSS) technology. CSS technology follows a standardized workflow that includes: (1) formulation development based on the physicochemical properties of the API; (2) formulation preparation and validation of the mixing and dosing steps; (3) 3D printing of dosage forms using optimized parameters; (4) packaging of the printed units; (5) quality control analysis and batch documentation; (6) technology transfer to end users, such as pharmacies or hospital facilities; and (7) implementation and customization of the system based on specific customer needs.

CSS technology follows a structured and scalable workflow (Figure 1) designed to ensure quality, reproducibility, and flexibility in personalized medicine production.^14–16^ The process begins with the development of drug formulations, guided by the physicochemical properties of the API such as solubility, melting point, and stability. Following this process, the formulation is prepared and validated through optimization of mixing and dosing parameters, ensuring uniformity and process robustness. The validated formulation is then loaded into the printer syringe, and dosage units are printed using customized settings tailored to the specific formulation and dosage form. After printing, products undergo packaging suitable for unit-dose or patient-specific delivery, followed by comprehensive quality control (QC) analyses, including weight, uniformity, and documentation for traceability. The final stages involve the technology transfer to end users, such as pharmacies or hospital facilities, and the adaptation and implementation of customer-specific needs, ensuring alignment with clinical workflows and regulatory standards.

### Water activity measurements

Water activity (a_w_) of formulations was measured to assess the potential for microbial growth and chemical stability. The test was conducted using a calibrated water activity meter (AquaLab Series 3TE, USA). Each formulation sample was placed in a clean, dry sample cup and loaded into the instrument chamber. Measurements were performed at room temperature (25 ± 2°C). The instrument was allowed to equilibrate until a stable reading was obtained, within 5–10 min per sample. All measurements were conducted in triplicate, and the mean values were recorded.

#### 1.5% ondansetron HCl dihydrate in CuraBlend^®^ gel tablet

The gel tablet containing 1.5% ondansetron HCl dihydrate was prepared by weighing 1.5% (w/w) ondansetron HCl dihydrate (Ph.Eur.) (ondansetron HCL⋅2H₂O), (Dr Reddy's, Hyderabad, India) and 1% (w/w) polysorbate 80 (Ph.Eur.) (Caelo, Hilden, Germany). Then 97.5% (w/w) the CuraBlend^®^ gel excipient base (CurifyLabs Oy, Helsinki, Finland) was mixed with 1.5% ondansetron HCl dihydrate and 1% (w/w) polysorbate 80 in the mixing jar (Gako Deutschland GmbH, Scheblitz, Germany). The excipients were mixed by the automated mixer, PM 140 planetary mixer (Gako Deutschland GmbH, Scheblitz, Germany) for 10 min at 2800 rpm.

#### 0.5% ondansetron HCl dihydrate in anhydrous troche

Before weighing any ingredients, the water bath set at +60 to +70 °C. A jar containing CuraBlend^®^ anhydrous troche excipient base (CurifyLabs Oy, Helsinki, Finland) was kept in the water bath at +60 to 70 °C for 1 h or until liquefied. The formulation was mixed gently with a spatula to ensure homogeneity. The jar containing 0.5% (w/w) ondansetron HCl dihydrate and 99.5% (w/w) of the excipient base was kept in water bath for 5 min. Then API and excipient base in the jar were mixed for 2 min at 2800 rpm by the PM 140 planetary mixer.

#### 0.25% and 0.5% ondansetron HCl dihydrate in ODF

The ODF formulations containing 0.25% and 0.5% ondansetron HCl dihydrate was started by weighing 0.25% (w/w) and 0.5% (w/w) ondansetron HCl dihydrate, 99.75% (w/w), and 99.5% (w/w) CuraBlend^®^ ODF excipient base (CurifyLabs Oy, Helsinki, Finland) respectively. Then the API and the excipient base were mixed by PM 140 planetary mixer for 2 min at 2800 rpm.

### 3D printing base dosing

The compounding step of dosage forms was performed using the CSS platform, specifically the Pharma printer (CurifyLabs Oy, Helsinki, Finland) a 3D printing device based on SSE technology, equipped with a precision-controlled dispensing unit. Prior to production, individual dose options for each formulation are predefined in the system's proprietary software as part of the digital batch record creation and stored in the integrated formulation library. During compounding, the user can select the appropriate dosage form to be dispensed, allowing flexible and consistent production aligned with the predefined parameters.^14,16^

#### Printing process of 1.5% ondansetron HCl dihydrate gel tablets

The printing process was conducted by filling the PVC syringes of 100 mL capacity (CurifyLabs, Helsinki, Finland) with mixed 1.5% ondansetron HCl dihydrate in CuraBlend^®^ gel formulation for printing process. The printing temperature for CuraBlend^®^ gel base was 41°C. The gel tablets were printed by using the Pharma printer into the blister (3/16 inch Mini Medi-Cap^®^ Plus™ Blisters, MD425, MediDose Inc, Ivyland, PA, USA). The printed tablets solidify 5 min in room temperature (20°C to 25°C), or 1 min in the fridge (2°C to 8°C). The printed tablets in the blister were sealed properly and stored in controlled condition.

#### Printing process 0.5% ondansetron HCl in anhydrous troches

A syringe was filled with the mixed formulation containing melted 0.5% ondansetron HCl dihydrate formulation for the printing*.* The printing temperature for anhydrous troches was 60 ^o^ C. The tablets were printed using the Pharma printer directly into blisters, which were closed with the heat sealer and stored in controlled condition.

#### Printing process 0.25% and 0.5% ondansetron HCl dihydrate ODFs

Before printing the film formulation, the laboratory incubator was preheated to 41 °C for drying the films. A syringe was filled with a mixed formulation as described above in section 2.1.3. The printing temperature for ODF CuraBlend^®^ was 25°C. Printing was performed directly into the blister packaging. The printed films were kept in the incubator at 41°C for 2–4 h. The printed films in the blister were sealed properly and stored in controlled condition.

### Packaging and Sealing

The Pharma printer extruded the gel tablets, anhydrous troches, and ODF formulations containing ondansetron HCl dihydrate in different packages. The gel tablets and ODFs were extruded into MediDose blisters (3/16 inch Mini Medi-Cap^®^ Plus™ Blisters, MD425, MediDose Inc, Ivyland, PA, USA). The anhydrous troche base formulation printed into gako BLIST-Rx™ tray (E12, 13mm) (Gako International GmbH, Scheßlitz, Germany).

The gel tablets and ODFs were sealed manually within the blisters by Fil-Form "25" Template and Roll-E-ZY Press (Pieceto, MediDose Inc, Ivyland, PA, USA) to ensure their integrity and protection from contamination and water loss. The anhydrous troche base was extruded in Gako blister and sealed by Gako BLIST-Rx™ holder and BLIST-Rx™ (Gako International GmbH, Scheßlitz, Germany).

### Mass Uniformity

A mass uniformity test was conducted by printing CuraBlend^®^ gel tablet of varying sizes 200 mg, 300 mg, 400 mg, and 500 mg containing 3, 4.5, 6, and 7.5 mg ondansetron HCl dihydrate respectively. Mass uniformity of anhydrous troche was done by printing 200, 300, 400, and 500 mg tablets containing 1, 1.5, 2, and 2.5 mg ondansetron HCl dihydrate respectively. The ODFs formulations were printed in 325, 375, and 425 mg films containing 0.81–2.13 mg ondansetron HCl dihydrate, the target doses are shown in Table 3. For each tablet size, 25 tablets were printed to evaluate the printing accuracy across different tablet sizes with consistent drug content. The weight of the tablets was measured during the printing process using an integrated scale within the Pharma printer. The software applied deviation limits of ±5% for tablets weighing over 250 mg and ±7.5% for those under 250 mg, in accordance with the guidelines specified in Ph. Eur Chapter 2.9.5, "Uniformity of Mass of Single-Dose Preparation".^17^

### Quality control sample analysis

The sample analysis was conducted using a High-Performance Liquid Chromatography (HPLC) system (Thermo Scientific™ Vanquish system, Germering, Germany). This system was integrated with the Chromeleon™ Chromatography Data System software (Dionex Softron GmbH, Germering, Germany). The HPLC setup included a C18 column with dimensions of 4.6×100 mm and a particle size of 2.5 μm VanGuard FIT, Wilmslow, UK. Additionally, the system was equipped with Diode Array Detectors, specifically the Thermo Scientific Vanquish detector, also produced by Dionex Softron GmbH in Germering, Germany. The ondansetron HCl dihydrate in CuraBlend^®^ gel base and anhydrous troche formulation was conducted by utilizing a phosphate buffer (potassium phosphate monobasic, Thermo Fisher, Massachusetts, United States) and acetonitrile (70:30), flow rate 0.7 ml/min, wavelength 216 nm, injection volume 10 µL, run time 6 min, and column temperature 30°C. However, for analyzing ondansetron HCl dihydrate in ODF formulation, a combination of phosphate buffer, acetonitrile, and methanol (50:40:10) and run time 4 min was applied.

### Chemical stability and drug uniformity

Stability test was conducted on 400 mg CuraBlend^®^ gel tablets containing 1.5% (6 mg) ondansetron HCl dihydrate, 400 mg anhydrous troche containing 0.5% (2 mg) ondansetron HCl dihydrate, and 325 mg ODF containing 0.81 and 1.63 mg ondansetron HCl dihydrate. Six individual samples were prepared in appropriate flasks to achieve a final solution containing 50 ppm ondansetron HCl dihydrate. Long-term stability tests followed the ICH Q1 (R2) guidelines^18^ under controlled conditions. Visual inspection, chemical stability (assay test) and pH measurement were performed at 0, 1, and 3, months to demonstrate the stability of this API in the different concentrations, and base formulations.

To assess ondansetron HCl dihydrate content uniformity, four different doses of ondansetron HCl dihydrate: 3, 4.5, 6, and 7.5 mg were formulated in CuraBlend^®^ gel tablet excipient base and 1, 1.5, 2, and 2.5 mg ondansetron HCl dihydrate were formulated in anhydrous troche excipient base. In addition, the content uniformity of the drug in ODFs was examined by printing 0.81, 0.94, 1.06, 1.63, 1.88, and 2.13 mg ondansetron HCl dihydrate in the film formulation. Each tablet was dissolved in an appropriate volumetric flask (final concentration: 50 ppm), and water was added as the diluent. The samples were then placed in water bath at 50°C and thoroughly mixed to create a homogeneous solution. After cooling to room temperature, the solutions were filtered through 0.22 mm membrane filters (MontaMil^®^, Frisenette ApS, Knebel, Denmark) to obtain clear solution. These samples were analyzed using HPLC. Finally, Equation (1) was used to calculate the %assay of ondansetron HCl dihydrate in tablets and ODF. The acceptance value (AV), Equation (2) was calculated for each batch according to the European Pharmacopoeia guidelines ("2.9.40 Uniformity of dosage units," 2005).^19^

(1) $$\text{\%}\;Assay\;=\frac{Average\;area\;of\;Test\;sample\;}{Average\;area\;of\;Standard\;sample\;}\times \frac{Weight\;of\;standard\;(mg)}{Volume\;of\;diluent\;(mL)}\times \frac{Volume\;of\;diluent\;(mL)}{Weight\;of\;test\;samples\;(gm)}\times \frac{Present\;weight\;(gm)}{Theoritical\;dose\;(mg)}\times 100$$

(2) $$\begin{array}{rl} AV= & |M-X|+ks\\ M= & X,\;if\;\;98.5\;\leq X\leq \;101.5\\ M= & 98.5,\;if\;\;X\;<\;98.5\\ M= & 101.5,\;if\;\;X\;>\;101.5\\ K= & 2.4\\ S= & standard\;deviation \end{array}$$

### Blend homogeneity

The syringe homogeneity test commenced by individually filling the Pharma printer syringe with 80–100 grams of each formulation. The syringes were kept in the heating jacket of Pharma printer for different times to reflect various usage scenarios. Then, each formulation was printed in different time points to investigate if API sedimentation takes place. Time points of printing each formulation are shown in Table 1. For each formulation, seven to eight blisters were printed, from which the first, fourth, and last prints were stored for analysis, resulting in three sets of 25 samples. The printing temperatures were formulation-specific: 25°C for ODF, 41°C for gel tablet, and approximately 60°C for the anhydrous troche formulation.

**Table 1.** The time points when formulations were printed.

Formulation	First print	Fourth print	Last print
1.5% (6 mg) ondansetron HCl dihydrate in CuraBlend^®^ gel tablet	115min	127min	136min
0.5% (2 mg) ondansetron HCl dihydrate in CuraBlend^®^ anhydrous troche	16min	46min	58min
0.5% (2 mg) ondansetron HCl dihydrate in CuraBlend^®^ ODF	8 min	19 min	32 min
0.25% (1 mg) ondansetron HCl dihydrate in CuraBlend^®^ ODF	3min	13min	31min

**Figure 2.**
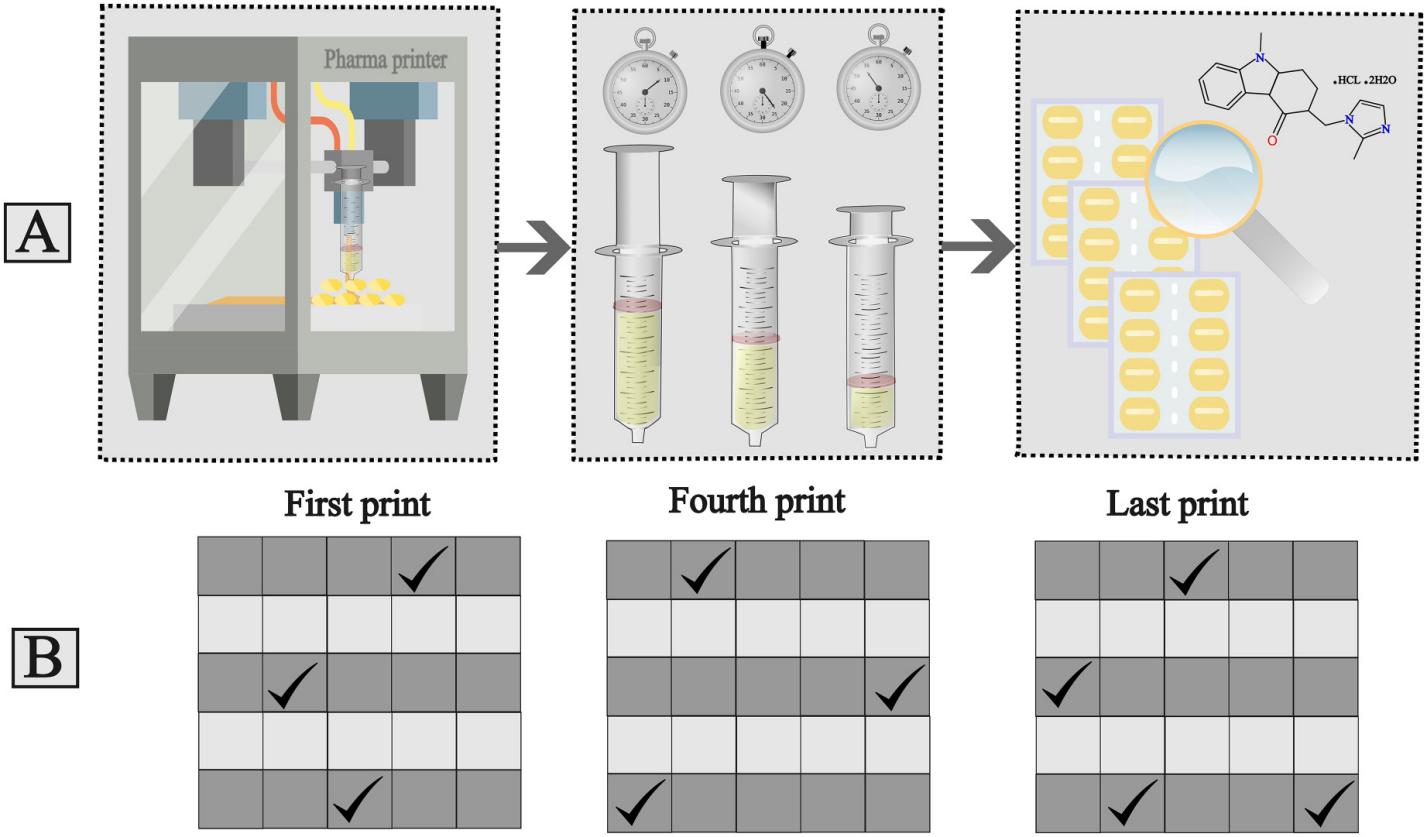
A. Syringe homogeneity test method, B. Randomly selected sample locations from blisters for quality control analysis.

For QC sample analysis, one sample (either tablets or ODFs) was taken from each grey row of the first and fourth prints. From the last print, one sample was taken from the first and second rows, and two samples were randomly selected from the last row, totaling 10 samples (Figure 2).

### Dissolution test

To conduct a dissolution study, the DT 128 dissolution tester (Erweka GmbH, Langen, Germany) was applied in accordance with the USP general methods. Dissolution test for ondansetron HCl dihydrate tablets (CuraBlend^®^ anhydrous troche and gel base) was conducted according to USP monograph for ondansetron HCl dihydrate tablet. The test for tablets was done in 500 ml de-ionized water as the medium, rotation speed 50 rpm, paddle (apparatus 2) and temperature 37± 0.5°C. The samples (1 mL) were withdrawn from each vessel (n=6) at 5, 10, 15, 20, 30, 45, and 60 min and 1 ml fresh medium (de-ionized water) were replaced instantly to compensate for the medium loss.

To perform dissolution study for ondansetron HCl dihydrate in CuraBlend^®^ ODF, the basket (apparatus 1), with rotation speed 100 rpm was utilized to demonstrate film formulation released profile. For dissolution study of film formulations, from each vessel (n=6) 1 mL samples were collected at 1, 3, 5, 10, 15, 20, and 30 min since CuraBlend^®^ ODF released much faster compared with tablets. Each sample was filtered by 0.22 mm membrane filters (MontaMil^®^, Frisenette ApS, Knebel, Denmark) to obtain the clear solution. The samples collected were analyzed by HPLC.

## Results

### Formulation development

**Figure 3.**
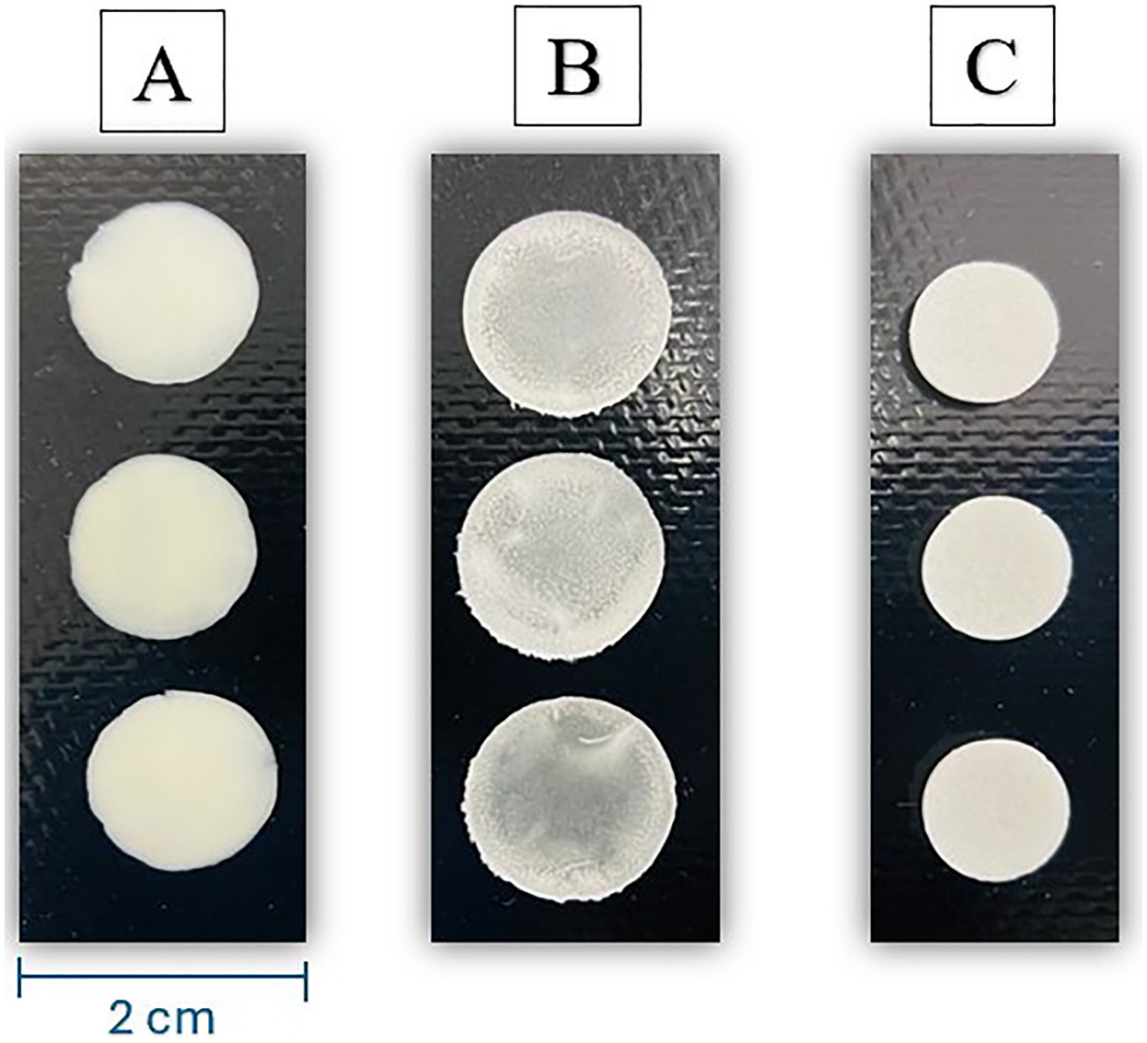
Appearance features of the different ondansetron hcl dihydrate formulations, A. 1.5% (6 mg) OND HCl dihydrate in CuraBlend^®^ gel tablet, B. 0.5% (1.63 mg) OND HCl dihydrate in CuraBlend^®^ ODF, C. 0.5% (2 mg) OND HCl dihydrate in CuraBlend^®^ anhydrous troche.

As illustrated in Figure 3, the three base formulations were utilized to print ondansetron HCl dihydrate using the Pharma printer. The CuraBlend^®^ gel tablet is an off-white, soft, chewable formulation with a vanilla flavor. The anhydrous (troche) formulation appears as an off-white, hard tablet with subtle vanilla and lemon flavors. The CuraBlend^®^ ODF (orally dissolving film) is a translucent white, flexible film characterized by a smooth surface, uniform thickness, absence of cracks, non-stickiness, and easy detachment from the blister packaging. The visual inspection of all three formulations was assessed over a three-month stability study.

### Water activity test

Table 2 demonstrates the water activity results for all three formulations.

**Table 2.** Active water in CuraBlend^®^ gel tablet, CuraBlend ^®^ anhydrous troche, and CuraBlend^®^ ODF excipient bases.

Formulation	Active water (n=3)	Temperature (n=3)
CuraBlend^®^ gel tablet	0.855	24.6 ^o^C
CuraBlend^®^ anhydrous troche	0.310	24.5 ^o^C
CuraBlend^®^ ODF	0.325	24.5 ^o^C

### Mass variation test of printed tablets and ODFs

**Figure 4.**
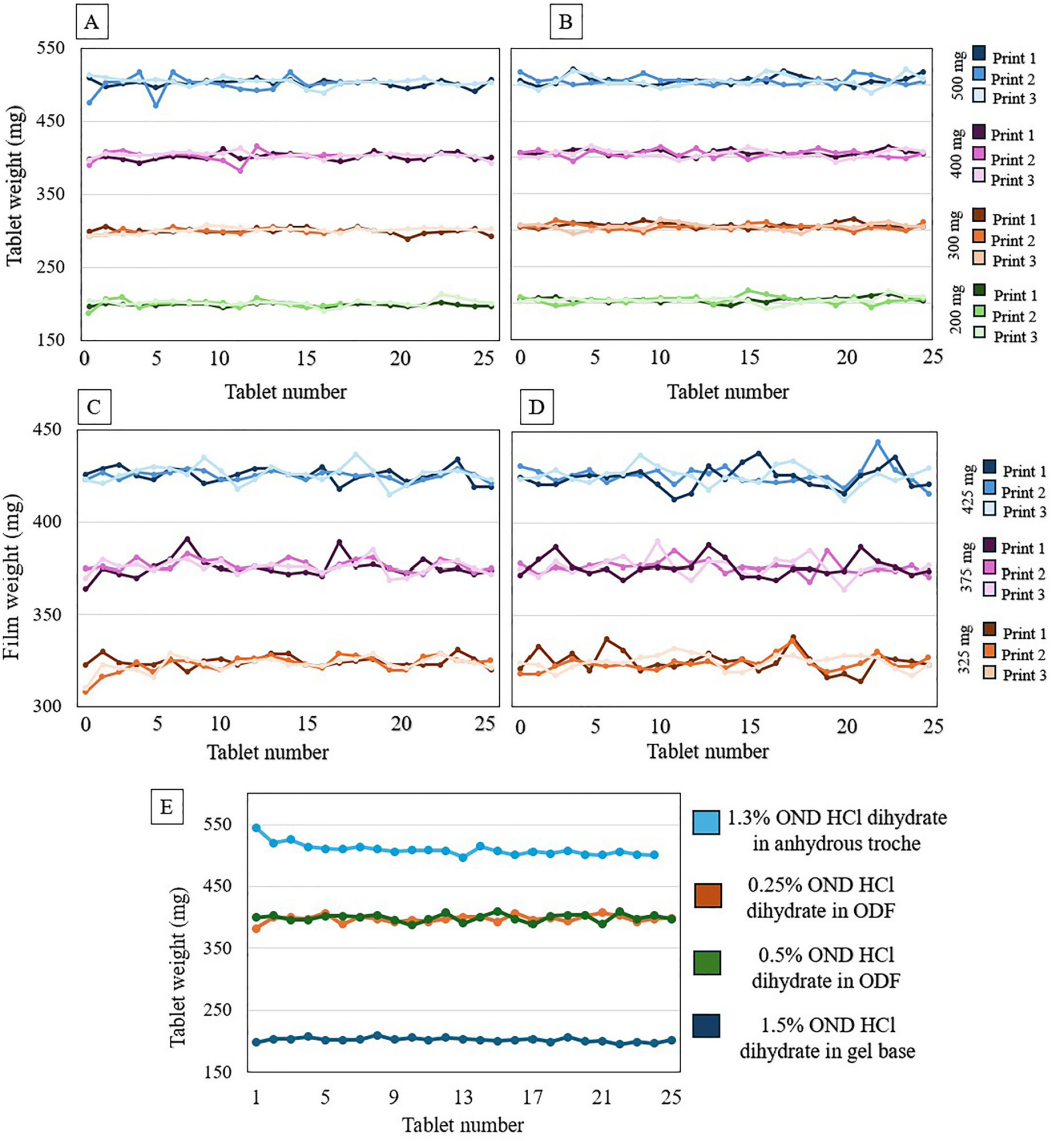
A. Mass uniformity of 1.5% (3–7.5 mg) ondansetron HCl dihydrate in CuraBlend^®^ gel tablets, B. Mass uniformity of 0.5% (1–2.5 mg) ondansetron HCl dihydrate in anhydrous troche, C. Mass uniformity of 0.25% (0.81–1.06 mg) ondansetron HCl dihydrate in CuraBlend^®^ ODF, D. Mass uniformity of 0.5% (1.63–2.13 mg) ondansetron HCl dihydrate in CuraBlend^®^ ODF, E. In-process controlled mass uniformity of different ondansetron HCl dihydrate dosage forms and concentrations which were printed in St Jude Children's Research Hospital.

The printer's mass variation accuracy test involved measuring the weights of CuraBlend^®^ gel tablets containing 1.5% ondansetron HCl dihydrate (3–7.5 mg) and anhydrous troches containing 0.5% ondansetron HCl dihydrate (1–2.5 mg) at four target tablet weights: 200 mg, 300 mg, 400 mg, and 500 mg. The target doses of ondansetron HCl dihydrate in CuraBlend^®^ gel tablet and anhydrous troche are presented in Table 3. Each target weight was printed 3 times (using 3 different batches) to demonstrate the repeatability of printing (Figure 4(A) and (B)).

**Table 3.** Target doses of ondansetron HCl dihydrate in CuraBlend^®^ gel tablets, anhydrous troches, and ODFs.

Tablets	200 mg printed tablet weight	300 mg printed tablet weight	400 mg printed tablet weight	500 mg printed tablet weight
1.5% ondansetron HCl dihydrate in CuraBlend^®^ gel tablet	3 mg OND	4.5 mg OND	6 mg OND	7.5 mg OND
0.5% ondansetron HCl dihydrate in CuraBlend^®^ anhydrous troche	1 mg OND	1.5 mg OND	2 mg OND	2.5 mg OND
Films	325 mg printed film	375 mg printed film		425 mg printed film
0.5% ondansetron HCl dihydrate in CuraBlend^®^ ODF	1.63 mg OND	1.88 mg OND		2.13 mg OND
0.25% ondansetron HCl dihydrate in CuraBlend^®^ ODF	0.81 mg OND	0.94 mg OND		1.06 mg OND

The mass variation test of CuraBlend^®^ ODFs containing 0.25% (0.81–1.06 mg) and 0.5% (1.63–2.13 mg) ondansetron HCl dihydrate were printed in 3 target weight groups 325, 375, and 425 (Figure 4(C) and (D)). Moreover, Figure 4(E) shows the mass uniformity of printed tablets and ODFs in different concentrations and dosage forms at the hospital setting. The CuraBlend^®^ gel tablet group consisted of 66 tablets, anhydrous troche group of 72 tablets, and CuraBlend^®^ ODFs 75 films each. The minimum and maximum weights, standard deviation and printing accuracy are shown in Table 4.

**Table 4.** Mass variation and printing accuracy of different ondansetron HCl dihydrate concentration in different dosage forms.

Product description	Target weight of tablet/film (mg)	Target dose (mg)	No. tablets	Minimum weight (mg)	Maximum weight (mg)	Mean weight (mg)	Standard deviation	Printing accuracy
1.5% ondansetron HCl dihydrate inCuraBlend^®^ gel tablet	200	3	66	187	213	200.1	4.2	100%
	300	4.5	66	289	308	300.2	3.6	100%
	400	6	66	382	416	402.4	5.2	100%
	500	7.5	66	471	517	502.0	7.8	98%
0.5% ondansetron HCl dihydrate inCuraBlend^®^ anhydrous troche	200	1	72	192	217	204.3	4.4	97%
	300	1.5	72	295	316	305.4	4.3	99%
	400	2	72	394	416	405.4	4.9	100%
	500	2.5	72	489	522	505.6	6.6	100%
0.5% ondansetron HCl dihydrate in CuraBlend**^®^** ODF	325	1.63	75	314	338	324.4	4.6	100%
	375	1.88	75	364	390	324.4	4.6	100%
	425	2.13	75	412	444	425.4	5.4	100%
0.25% ondansetron HCl dihydrate inCuraBlend^®^ ODF	325	0.81	75	308	331	323.7	3.9	99%
	375	0.94	75	364	391	376.0	4.2	100%
	425	1.06	75	415	437	425.6	3.8	100%

Table 5 demonstrates the final CuraBlend^®^ ODFs weights, deviation, and ratio of dried weight to weight after incubation. The average dried film weights were 105 mg, 120 mg, and 135 mg, respectively, 0.32% as ratio of dried weight/wet weight (%) and relative standard deviations (RSDs) decreasing as the initial weight increased, 1.56% for 325 mg, 0.42% for 375 mg, and 0.34% for 425 mg films, suggesting improved reproducibility at higher masses.

**Table 5.** Average dried weights of CuraBlend^®^ ODFs after water evaporation during incubator drying.

Wet weights	325 mg CuraBlend^®^ ODF	375 mg CuraBlend^®^ ODF	425 mg CuraBlend^®^ ODF
Average weight after water evaporation (mg)	105 mg	120 mg	135 mg
RSD (%)	1.56	0.42	0.34
Ratio of dried weight/wet weight (%)	0.32	0.32	0.32

### Stability and content uniformity

Table 6 presents ambient stability (visual inspection and chemical stability) and dissolution test results for different concentrations of ondansetron HCl dihydrate for different formulations over a 3-month period. For each time point, six samples were used and analysised by HPLC.

**Table 6.** Stability of ondansetron HCl dihydrate in different formulations.

Product description	Zero-point (n=6)	1 Month (n=6)	3 Month (n=6)
1.5% (6 mg) ondansetron HCl dihydrate in CuraBlend^®^ gel base	Assay:105.2% pH:4.94 Dissolution: 60% in 15 min 99% in 60 min	Assay:108.1% pH:4.94 Dissolution: 55% in 15 min and 102% in 60 min	Assay: 103.3% pH: 4.98 Dissolution: 59% in 15 min and 105% in 60 min
0.5% (2 mg) ondansetron HCl dihydrate in CuraBlend^®^ anhydrous troche	Assay: 97.9% Dissolution: 107% in 15 min	Assay: 99.4% Dissolution: 97% in 15 min	Assay: 99.5% Dissolution: 94% in 15 min
0.5% (1.63 mg) ondansetron HCl dihydrate in CuraBlend^®^ ODF	Assay: 94.3% pH: 3.61 Dissolution: 95% in 3 min and 103% in 5 min	Assay: 99.1% pH: 3.69 Dissolution: 101% in 3 min and 105% in 5 min	Assay: 94.2% pH: 3.69 Dissolution: 97.7% in 3 min and 101.2% in 5 min
0.25% (0.81 mg) ondansetron HCl dihydrate in CuraBlend^®^ ODF	Assay: 100.6% pH: 3.64 Dissolution: 98% in 3 min and 102% in 5 min	Assay: 97.1% pH: 3.71 Dissolution: 95% in 3 min and 105% in 5 min	Assay: 95.9% pH:3.69 Dissolution: 88% in 3 min and 100.8% in 5 min

**Figure 5.**
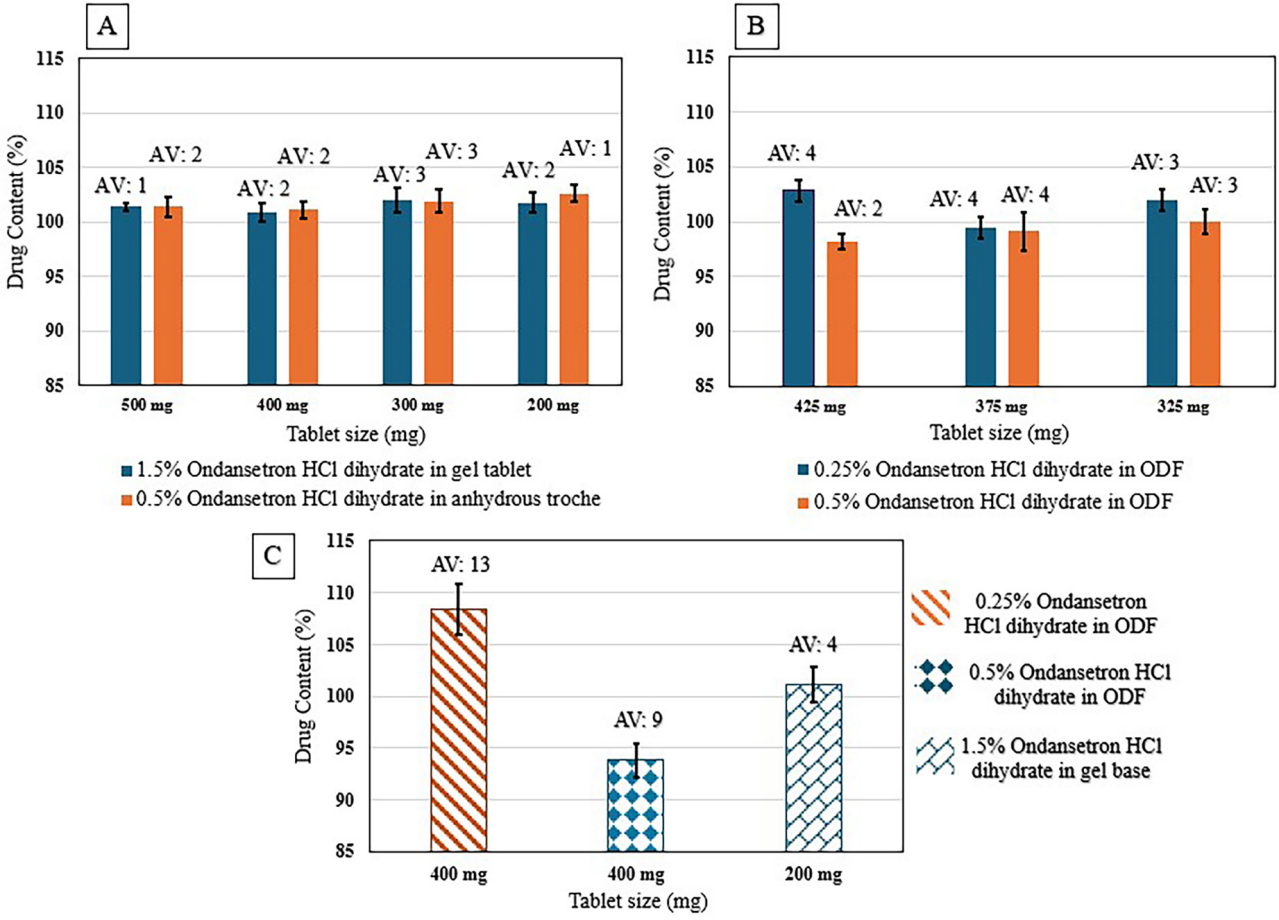
A. Content uniformity of 1.5% (3–7.5 mg) ondansetron HCl dihydrate in CuraBlend^®^ gel tablets and 0.5% (1–2.5 mg) ondansetron HCl dihydrate in anhydrous troche, B. Content uniformity of 0.25% (0.81–1.06 mg) and 0.5% (1.63–2.13 mg) ondansetron HCl dihydrate in CuraBlend^®^ ODF, C. Content uniformity of printed tablets/films in St Jude hospital.

The content uniformities of SSE tablets (with target doses of 3–7.5 mg ondansetron HCl dihydrate in CuraBlend^®^ gel tablet and 1–2.5 mg ondansetron HCl dihydrate in CuraBlend^®^ anhydrous troches were determined for four different sizes, 200 mg, 300 mg, 400 mg, and 500 mg (Figure 5(A)). In addition, ondansetron HCl dihydrate uniformity in CuraBlend^®^ ODF (325, 375, and 425 mg) with different target doses (0.81–2.13 mg ondansetron HCl dihydrate) were presented in Figure 5(B). Formulations of 1.5% ondansetron HCl dihydrate in 200 mg CuraBlend^®^ gel tablets (containing 3 mg ondansetron HCl dihydrate), as well as 0.25% and 0.5% ondansetron HCl dihydrate in CuraBlend^®^ ODFs (containing 1 mg and 2 mg ondansetron HCl dihydrate, respectively), were prepared for method and product validation in a hospital setting at St Jude Children's Research Hospital, Memphis, Tennessee, USA (Figure 5(C)).

### Blend homogeneity

The Table 7 presents the average content uniformity of printed tablets and films, standard deviation, and acceptance value, highlighting the homogeneity of the formulations in Pharma printer's syringe throughout the print cycle.

**Table 7.** The syringe homogeneity results of ondansetron HCl dihydrate formulations.

Product description	Print 1	Print 2	Print 3	Average (n=10)	RSD (%)	AV
1.5% (6 mg) ondansetron HCl dihydrate in gel tablet**^®^**	115 min: 101.9%	127 min: 102.6%	136 min: 102.6%	102.4%	0.77	3
0.5% (2 mg) ondansetron HCl dihydrate in anhydrous troche	16 min: 102.2%	46 min: 103.6%	58 min: 104.4%	103.4%	1.53	6
0.5% (2 mg) ondansetron HCl dihydrate in CuraBlend^®^ ODF	8 min: 99.1%	19 min: 100.3%	32 min: 100.1%	99.8%	1.46	3
0.25% (1 mg) ondansetron HCl dihydrate in CuraBlend^®^ ODF	3 min: 101.7%	13 min: 100.0%	31 min: 101.9%	101.2%	2.19	5

### In-Vitro dissolution study

**Figure 6.**
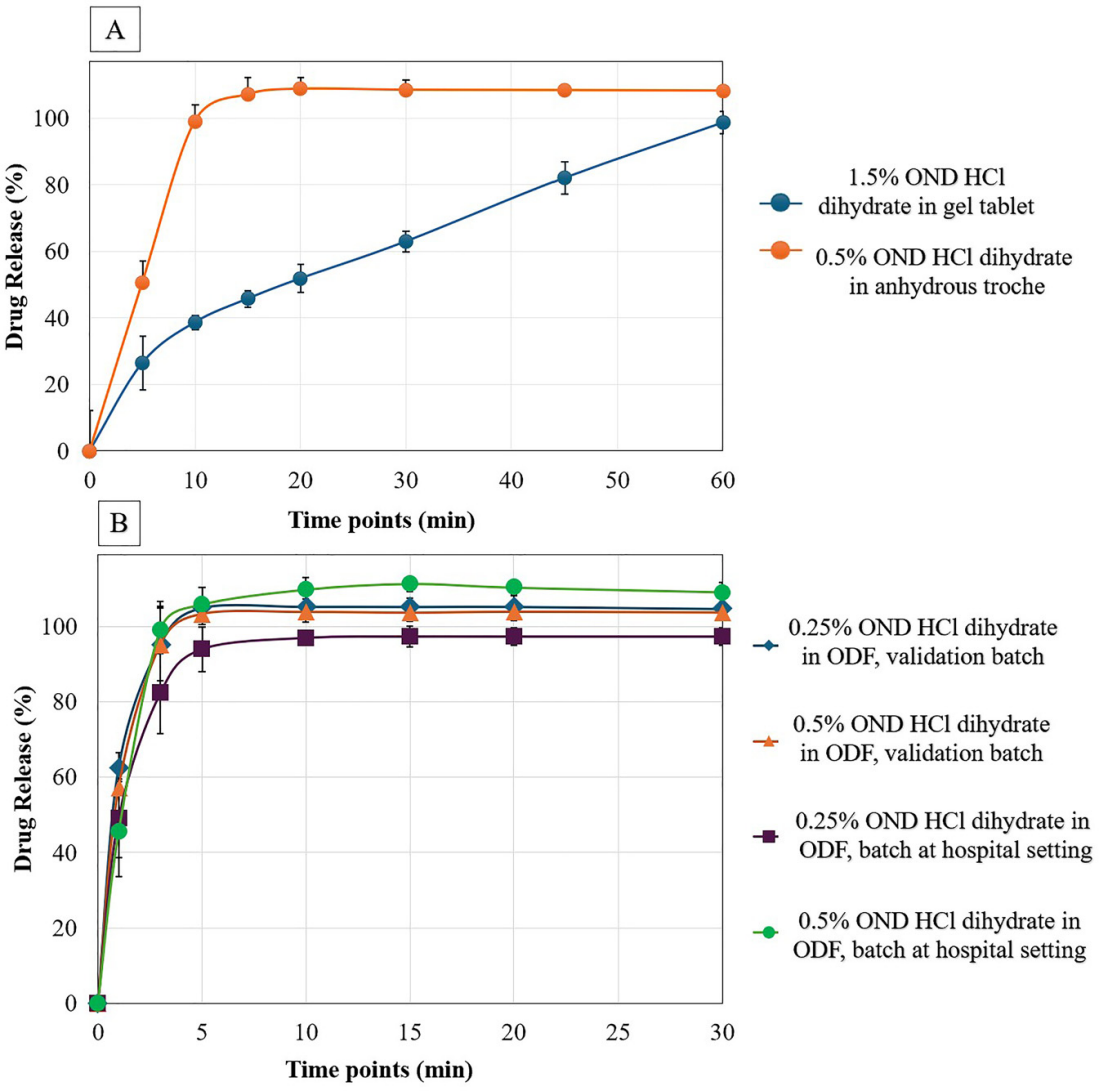
A. In-Vitro Dissolution profile of 1.5% (6 mg) ondansetron HCl dihydrate in gel tablet and 0.5% (2 mg) ondansetron HCl dihydrate in anhydrous troche, B. In-Vitro dissolution profile of 0.25% (0.81 mg and 1mg), 0.5% (1.63 mg and 2 mg) ondansetron HCl dihydrate in ODF validation batch and batch at hospital setting respectively.

In-vitro dissolution profile of 1.5% (6 mg) ondansetron HCl dihydrate in CuraBlend^®^ gel tablet and 0.5% (2 mg) ondansetron HCl dihydrate in anhydrous troche formulations are demonstrated in Figure 6(A). The dissolution study of tablets was assessed at 0, 5, 10, 15, 20, 30, 45, and 60 min. The in-vitro dissolution data for anhydrous troche demonstrated approximately 100% of ondansetron HCl dihydrate was released within 10 min. This complies with the USP Pharmacopeia Test 1, which requires Q > 80% in 15 min.^20^ However, the 1.5% (6 mg) of ondansetron HCl dihydrate in CuraBlend^®^ gel tablet formulation released within 60 min. Although the chewable tablet did not meet the USP dissolution criteria, it is important to note that this formulation is designed for chewing. In real-world use, the mechanical action in the mouth would significantly accelerate drug release compared to the conditions in a standard dissolution tester.

The CuraBlend^®^ ODF containing 0.25% (0.81 mg and 1mg), 0.5% (1.63 mg and 2 mg) ondansetron HCl dihydrate as validation batch and batch at hospital setting respectively were conducted at 0, 1, 3, 5, 10, 15, 20, and 30 min (Figure 6(B)). The formulation prepared as validation batch are compared with samples prepared in St Jude Children's Research Hospital (batch in hospital setting). The released drug profiles demonstrated that 100% of drug released within 3 min which is according to ODF acceptance criteria in USP.

## Discussion

This study demonstrates the application of 3D printing-based dispensing as a pharmaceutical compounding solution for manufacturing various dosage forms with different strengths of ondansetron HCl dihydrate. Formulation development and process validation was performed prior to compounding and test printing was carried out at St Jude Children's Research Hospital. Successful process transfer highlights the accuracy and consistency of the approach in on-site production. The results confirm the system's ability to produce precise, reproducible, and stable pharmaceutical products. These findings, supported by comprehensive quality control analyses, underscore the potential of this technology to modernize manual compounding practices and advance personalized medicine in both hospital and commercial pharmacy settings, ultimately improving patient care.

Personalizing ondansetron HCl dihydrate for pediatrics has been investigated.^4,21,22^ Precise, individualized dosing helps ensure optimal effectiveness while minimizing side effects, such as drowsiness or cardiac risks like QT prolongation.^23^ Additionally, personalized forms like chewable,^24^ disintegrating tablets or ODFs^11^ can improve compliance and comfort, especially for younger children who may struggle with swallowing hard pills.^16^

Mass variation results indicated the weight consistency and printing accuracy of various ondansetron HCl dihydrate formulations, including gel base, anhydrous troche, and ODFs in different drug concentrations (0.81–7.5 mg). In this study, an integrated balance recorded the tablets or ODFs weight during manufacturing process. The software applies an acceptance criterion according to European Pharmacopeia (Ph. Eur.) 2.9.5,^17^ a deviation limit of ±5% to tablets weighing over 250 mg, and ±7.5% to those under 250 mg. Each formulation had different target weights ranging from 200 mg to 500 mg for gel base CuraBlend^®^ (3–7.5 mg ondansetron HCl dihydrate) and anhydrous troche (1–2.5 mg ondansetron HCl dihydrate), and 325 mg to 425 mg for CuraBlend^®^ ODFs (0.81–2.13 mg ondansetron HCl dihydrate). However, due to possible air pockets in the syringe during printing^25^ the printing accuracy in anhydrous troche formulation is lower than other formulations. In the real world, the tablets or ODFs out of these limits can be ignored and replaced with the tablets or films in the accepted range.

The mean weights of printed drug products closely match the target weights, with low standard deviations. Printing accuracy is high, ranging from 97% to 100%, demonstrating accurate manufacturing processes. Table 5 demonstrates the final CuraBlend^®^ ODFs weights, deviation, and ratio of dried weight to wet weight after incubating CuraBlend^®^ ODFs in incubator at 41°C for 2–4 h. These results demonstrate that the drying process is highly consistent and scalable, with minimal variability at higher weights. The initial CuraBlend^®^ ODF formulation contained approximately 70% water by weight; consequently, during the drying process, around 70% of the total mass was lost due to water evaporation in incubating step. The results confirm that the drying process effectively removes the expected proportion of water which is essential for CuraBlend^®^ ODF stability over time. The uniformity in the dried-to-wet weight ratio supports the robustness of the formulation and drying method, making it suitable for reliable production of CuraBlend^®^ ODFs across varying batch sizes.

In a recent study, the use of Selective Laser Sintering (SLS) 3D printing has been investigated to create orally disintegrating tablets (ODTs)—called printlets—that contain ondansetron.^26^ Comparatively, SSE produces orally disintegrating tablets at lower temperatures, preserving drug stability for heat-sensitive APIs. Compared to SLS, SSE also ensures quicker disintegration and is more efficient for patient-friendly dosage forms. Thus, in the real world, SSE can be conducted as a compounding system solution for ondansetron manufacturing in different dosage forms and concentrations. In line with our study, recent pharmaceutical research developed a child-friendly, chewable ondansetron dosage form using SSE 3D printing and a gelatin-based formulation, successfully extruding gummies containing 4 mg of ondansetron. This approach not only ensured accurate dosing but also demonstrated the potential of SSE technology to produce personalized, palatable medications tailored for pediatric patients.^24^

The chemical stability test (assay) was conducted with room temperature samples. During the stability time points, printed CuraBlend^®^ gel tablets containing 1.5% (6 mg) ondansetron HCl dihydrate, remained an off-white, soft, chewable form with a vanilla flavor. The CuraBlend^®^ ODF formulations remained translucent white, flexible film characterized by a smooth surface and uniform thickness, free of cracks, non-sticky, easily detachable from the blister. It melted easily in the dissolution medium over stability test, ensuring rapid disintegration and effective delivery of the active pharmaceutical ingredient. Stability studies confirmed that these properties remained consistent under controlled storage conditions.^10,27^ Furthermore, anhydrous troche formulation containing 0.5% (2 mg) ondansetron HCl dihydrate was stable over 3 months. The printed tablets were off-white hard tablets with mild vanilla and lemon flavors. Supporting that ondansetron HCl dihydrate, is stable and has potency upon administration in this formulation. The achieved value complies with ICH Q1A (R2) guidelines.^18^

Acceptance value was obtained less than 4 for all of ondansetron HCl dihydrate concentrations printed as validation in various formulations. Acceptance value of less than 15 and low standard deviation for products printed at hospital setting indicated the uniformity of the drug in the formulations.^19^ In average, lower acceptance value for higher ondansetron HCl dihydrate concentrations demonstrated that higher API concentration contributes to better uniformity.^15,16,28^

The blend homogeneity data from the syringe tests for the Pharma printer indicate that the formulation is homogeneous within the syringe throughout the entire printing process. This uniformity is observed across different concentrations and types of formulations, ensuring reliable and reproducible results. This finding aligns with the recent study on corticosteroid products.^29^ The reported data is crucial for ensuring that all formulations in the Pharma printer's syringe contain the correct amount of active ingredient and for preventing issues such as drug sedimentation during the manufacturing process. The acceptance value which is critical to demonstrate drug uniformity obtained less than 15 which is complied with USP.^30^ This type of syringe homogeneity approach which has been described in the current article has been a request from the field, i.e., to test the uniformity of the formulation throughout the printing process before printing doses for patients.

These results are particularly relevant given the limitations of manual compounding methods, which remain prevalent in pediatric and hospital pharmacy settings. Manual approaches often lack consistent process controls and are subject to operator variability, which can lead to dose deviations and reduced reproducibility.^31^ By contrast, the SSE 3D printing platform used in this study ensures batch traceability, electronic batch records, and precise dispensing, reducing the risk of compounding errors and improving overall medication quality. This is especially important for vulnerable populations such as children and chemotherapy patients, where underdosing or inconsistent administration can lead to suboptimal therapeutic outcomes.^14,32,33^

In a recent study ondansetron HCl dihydrate was formulated in ODF formulation containing hydroxypropyl methylcellulose (HPMC), polyvinyl alcohol (PVA), citric acid, sorbitol, fructose, and/or polyethylene glycol (PEG) 400. The formulation containing polymer PVA showed a higher drug release compared with formulation containing HPMC. Since PVA has higher water hydration ability. Thus, this formulation released 92% in 5 min which was the highest drug released between all formulations.^34^ In the current study, ondansetron HCl dihydrate was formulated in ODF containing polyethylene oxide (PEO) and maltodextrin as polymers, and more than 50% of the drug was released within 1 min and approximately 95% within 3 min. These results show that the tested ODF formulation decreased time to drug release.

Water activity (a_w_) is a critical parameter in pharmaceutical formulation development, influencing microbial stability, chemical degradation, and overall shelf-life. In this study, three dosage forms CuraBlend^®^ gel tablet, CuraBlend^®^ anhydrous troche, and CuraBlend^®^ ODF were evaluated for their water activity at room temperature. The CuraBlend^®^ gel tablet exhibited a high water activity value of 0.855, which exceeds the commonly accepted threshold of 0.6 for microbial safety in solid oral dosage forms base on USP <1112>. However, this formulation contains potassium sorbate, a widely used antimicrobial preservative, which helps mitigate the risk of microbial growth. In contrast, the CuraBlend^®^ anhydrous troche and CuraBlend^®^ ODF showed significantly lower water activity values of 0.310 and 0.325, respectively. These values fall well below the microbial growth threshold, indicating better stability and reduced risk of microbial contamination. The low water activity in these formulations is particularly advantageous for pediatric applications, where safety and shelf-life are critical.

The successful demonstration of process validation, stability, and dose accuracy also strengthens the regulatory case for adopting automated 3D printing technologies in clinical environments. As regulatory bodies increasingly call for standardization, traceability, and data integrity in compounding practices, systems like the Pharma printer provide a viable pathway for compliance with USP <795>, <905>, and ICH Q1A(R2) guidelines.^30,35,36^ Furthermore, integration into hospital settings aligns with initiatives to decentralize manufacturing and reduce reliance on commercially available products that may not meet individual patient needs. The technology also opens the door for formulary expansion, allowing institutions to offer age-appropriate, dose-flexible, and palatable alternatives tailored to local patient populations.

In pediatric care, treatment adherence is often compromised by factors such as dysgeusia (unpleasant taste), oral aversion, and difficulty swallowing conventional solid dosage forms.^37^ These challenges are particularly pronounced in therapies involving bitter APIs like ondansetron HCl dihydrate, which may trigger gag reflexes or lead to refusal of treatment.^38^ Such adherence issues are not merely inconvenient but can contribute to underdosing, reduced therapeutic efficacy, and increased risk of treatment failure. The use of chewable gel tablets and ODF developed in this study addresses these concerns by offering palatable, easy-to-administer alternatives that dissolve rapidly in the mouth or require minimal effort to ingest. Application of film formulation as a solution for ondansetron HCl dihydrate formulation to avoid bitter taste and bitterness has been investigated before.^12,34^ The incorporation of child-friendly flavors and adaptable textures improves acceptability and can play a crucial role in maintaining therapeutic regimens—especially in settings where repeated dosing is required over several days, such as post-chemotherapy antiemetic prophylaxis.

In general, in-vitro dissolution profile of each product depends on the shape, thickness, and the tablet diameter, the solubility of API, the selected formulation, and the dissolution medium.^39^ In this study, the in-vitro dissolution of CuraBlend^®^ gel tablet (chewable), anhydrous troche, and ODF were conducted. The dissolution medium for all 3 dosage forms of ondansetron HCl dihydrate are same. Thus, the differences in dissolution profiles of these three products are related to the formulation. The formulation for CuraBlend^®^ gel tablet containing a significant portion of gelatin which is prone to cross-linking, causes slowing down the dissolution process.^40^ However, in anhydrous troche formulation, PEG 1500 can improve solubilization properties and ability to enhance the dissolution rate of drugs. It reduces the crystallinity of the drug, leading to a faster dissolution.^41^ CuraBlend^®^ gel tablet are easier to administer, especially for children and elderly patients. However, slower drug release of ondansetron HCl dihydrate was observed in gel tablet. Additionally, we should highlight that chewing the tablet can help in the initial breakdown of the placebo containing drug, potentially enhancing drug bioavailability to compensate for any delay in drug release.^42^

This research contributes to the growing evidence that digitally controlled, automated compounding platforms can meet the complex requirements of pediatric pharmacotherapy. By leveraging SSE based 3D printing within a compounding system framework, hospital pharmacies can produce customized ondansetron HCl dihydrate dosage forms that address taste, dosing accuracy, and patient usability advancing both clinical outcomes and operational efficiency in pediatric care.

In this study, to demonstrate the feasibility of SSE for personalized medicine, standardized doses (0.81–7.5 mg ondansetron HCl dihydrate) were used as ‘case studies’ across three dosage forms: CuraBlend^®^ gel tablets, anhydrous troches, and ODFs. This approach enabled evaluation of print quality, content uniformity, in-vitro dissolution, and compliance with pharmacopeial standards. However, the primary advantage of this technology lies in its ability to manufacture patient-specific dosages tailored to individual needs such as age, weight, and therapeutic requirements. The technology is already in use at hospital settings and enables on-demand production of customized formulations. Additional quality control testing ensures the safety and efficacy of printed tablets, films, and troches for individual patients.

In contrast with other 3D printing technologies such as fused deposition modeling (FDM) and SLS, which require specialized equipment, expertise, and heat-resistant APIs and other excipients, SSE offers a more practical, time-saving and accessible solution for producing oral dosage forms. The technology described here is based on validated and standardized methods to produce compounded drugs with integrated quality control, SSE technology has already shown strong potential for implementation in hospital pharmacies and clinical compounding units, enabling on-demand production of personalized medications. This is already reality in both in Europe and US due to its practical nature and capacity to automate and standardize manual compounding contributing also to increased quality of compounded products.

Beyond pediatric applications, SSE 3D printing holds promise across broader clinical settings where timely access to personalized formulations is essential. Fields like oncology and pediatrics benefit from precise dose, and time-saving production methods. Ongoing developments include scaling these solutions across pharmacies and hospital networks by linking electronic prescriptions directly to production systems and integrating with pharmacy modules in Electronic Health Records (EHRs) for automatic documentation, and embedding real-time quality assurance using process analytical technology tools such as NIR or Raman spectroscopy. These advancements could significantly ease pharmacists’ workload while supporting more agile, patient-centered care.

## Conclusion

This research highlighted the effectiveness and precision of the Pharma printer in manufacturing various dosage forms of ondansetron HCl dihydrate. The various ondansetron HCl dihydrate doses and formulations were validated using the SSE 3D Pharma printer and validated by quality control data. In the subsequent phase, the selected formulations and dosages underwent the printing process at St Jude Children's Research Hospital utilizing the validated Pharma printer. The results demonstrated the printer's capability to consistently produce stable pharmaceutical products with precise mass uniformity and high printing accuracy across diverse dosage forms, including gel tablets, anhydrous troches, and ODFs. The comprehensive quality control tests including stability, drug uniformity, blend homogeneity during printing, and in-vitro dissolution studies underscore the reliability of the compounding system solution paving the way for personalized medicine by modernizing manual compounding methods and enhancing patient care in hospital and commercial pharmacies.

## Abbreviations

API: Active Pharmaceutical Ingredient
CSS: Compounding System Solution
SSE: Semi-solid extrusion
3D: Three-dimension
SLS: Selective Laser Sintering
OND: ondansetron
HCl: Hydrochloride
HPLC: High-Performance Liquid Chromatography
NIR: Near-infrared
PAT: Process Analytical Technology
ODF: Orodispersible Film
AV: Acceptance Value
HPMC: Hydroxypropyl Methylcellulose
PVA: Polyvinyl Alcohol
PEG: Polyethylene glycol
PEO: polyethylene oxide
CMC: Carboxymethylcellulose
CU: Content Uniformity
Ph. Eur.: European Pharmacopoeia
RSD%: Relative Standard Deviation (Percentage)
SD: Standard Deviation
USP: United States Pharmacopeia
FDM: Fused Deposition Modeling
